# Reducing child global undernutrition at scale in Sofala Province, Mozambique, using Care Group Volunteers to communicate health messages to mothers

**DOI:** 10.9745/GHSP-D-12-00045

**Published:** 2013-03-21

**Authors:** Thomas P Davis, Carolyn Wetzel, Emma Hernandez Avilan, Cecilia de Mendoza Lopes, Rachel P Chase, Peter J Winch, Henry B Perry

**Affiliations:** a Food for the Hungry, Washington, DC, USA; b International Relief and Development, Beira, Mozambique; c FHI 360, Beirea, Sofala, Mozambique; d Department of International Health, Johns Hopkins Bloomberg School of Public Health, Baltimore, MD, USA Correspondence to Henry Perry, heperry@jhsph.edu

## Abstract

**Background:**

Undernutrition contributes to one-third of under-5 child mortality globally. Progress in achieving the Millennium Development Goal of reducing under-5 mortality is lagging in many countries, particularly in Africa. This paper shares evidence and insights from a low-cost behavior-change innovation in a rural area of Mozambique.

**Intervention:**

About 50,000 households with pregnant women or children under 2 years old were organized into blocks of 12 households. One volunteer peer educator (Care Group Volunteer, or CGV) was selected for each block. Approximately 12 CGVs met together as a group every 2 weeks with a paid project promoter to learn a new child-survival health or nutrition message or skill. Then the CGVs shared the new message with mothers in their assigned blocks.

**Methods of evaluation:**

Household surveys were conducted at baseline and endline to measure nutrition-related behaviors and childhood nutritional status.

**Findings:**

More than 90% of beneficiary mothers reported that they had been contacted by CGVs during the previous 2 weeks. In the early implementation project area, the percentage of children 0–23 months old with global undernutrition (weight-for-age with z-score of less than 2 standard deviations below the international standard mean) declined by 8.1 percentage points (*P*<0.001), from 25.9% (95% confidence interval [CI] = 22.2%–29.6%) at baseline to 17.8% at endline (95% CI = 14.6%–20.9%). In the delayed implementation area, global undernutrition declined by 11.5 percentage points (*P*<0.001), from 27.1% (95% CI = 23.6%–30.6%) to 15.6% (95% CI = 12.6%–18.6%). Total project costs were US$3.0 million, representing an average cost of US$0.55 per capita per year (among the entire population of 1.1 million people) and US$2.78 per beneficiary (mothers with young children) per year.

**Conclusion:**

Using the Care Group model can improve the level of global undernutrition in children at scale and at low cost. This model shows sufficient promise to merit further rigorous testing and broader application.

## Abstract (precis)

Care Group peer-to-peer behavior change communication improved child undernutrition at scale in rural Mozambique and has the potential to substantially reduce under-5 mortality in priority countries at very low cost.

## INTRODUCTION

The United Nations Children's Fund (UNICEF) and the World Health Organization (WHO), together with more than 40 countries around the world, are now calling for a renewed commitment to child survival to eliminate preventable child deaths by the year 2035.^1-2^ This will require more than doubling the global annual rate of decline in the under-5 mortality rate, from 2.5% in the previous decade to 5.3%.^3^ Health programs will need to place a new emphasis on reaching the most marginalized populations where child mortality rates are currently the highest, with a priority on changing household behaviors, particularly those that are nutrition-related. The most recent progress assessment finds that only 4 of 42 priority countries in sub-Saharan Africa are on track to achieving Millennium Development Goal 4 (MDG 4) for child health (reducing the under-5 mortality rate by two-thirds between 1990 and 2015).^4^

Undernutrition in mothers and children is an important underlying contributor to an estimated 35% of child deaths and responsible for 11% of the global disease burden.^5^ Although many developing countries have improved coverage of several key child-survival interventions, there has been less progress in coverage of key interventions for improving levels of childhood nutrition, particularly in Africa.^4^ One recent analysis found virtually no improvement in child growth based on anthropometric indicators between 1985 and 2011 in sub-Saharan Africa.^6^

Poor nutrition contributes to about one-third of child deaths.

In Mozambique, 44% of children under 5 years old are stunted, and 18% are underweight.^7^ Diarrhea is a contributor to malnutrition, and malnutrition aggravates the severity of, and mortality from, diarrhea.^8^ In Mozambique, 10% of under-5 deaths are due to diarrhea.^4^ The country has one of the highest under-5 mortality rates in the world, at 153 deaths per 1,000 live births, and it is not on track to achieve MDG 4 in the near future.^4^ Recent at-scale, community-based programs in Africa have shown disappointing results in improving coverage of child-survival interventions that are based on changes in household practices, such as nutrition- and diarrhea-related indicators.^9^

Determining the best ways to improve practices for preventing and treating diarrhea and for improving childhood nutrition at scale remains a key challenge for Africa. We have identified only 2 sub-Saharan African programs that cover populations of more than 1 million people and are aimed at improving child-nutrition practices that do not involve food supplementation. The first did not measure changes in childhood nutritional status,^9^ and the second did not observe any improvements that could be attributed to the interventions.^10^

We developed and tested a service model that uses only 6.6 paid staff per 100,000 people and that relies on community volunteers. This paper reports on changes in coverage of nutrition- and diarrhea-related interventions and in childhood nutritional status in 7 districts in Sofala Province, Mozambique, compared with changes reported for Mozambique nationally in the national Demographic and Health Surveys (DHS).

## METHODS

### Project Context

The project was carried out by Food for the Hungry/Mozambique (FH/M), in collaboration with the Ministry of Health and with additional technical support from staff at Food for the Hungry's Global Service Center. The project took place from 2005–2010 in 7 districts of Sofala Province, which have a combined population of 1.1 million people. During the first 2.5 years, the project implemented services in 4 districts (Caia, Chemba, Manga, and Marringue) with 42% of the total project population (Area A). During the final 2.5 years, the project expanded to an additional 3 districts (Dondo, Gorongosa, and Nhamatanda) with another 58% of the project population (Area B).

FH/M selected the project area because it had high levels of malnutrition and low coverage of key child-survival interventions. The area is almost entirely rural, and many villages are at considerable distance from a health facility. Residents are primarily subsistence farmers with small plots of land. Motorized transport in the form of motorcycles and vehicles is limited. During the project period, there were no community-level health workers trained by the government and no other nongovernmental organizations (NGOs) working in nutrition in the project areas. There were 46 well-staffed and well-attended health posts and health centers in the 4 start-up districts. Findings from the baseline household survey (described below) showed that 50% of women with a child 0–23 months old had never attended school, 35% had obtained some primary-level schooling, and 15% had obtained 6 or more years of education.

### Intervention

FH/M implemented the Care Group intervention model as described in *The Care Group Difference* manual published by World Relief and the CORE Group.^11^

With the help of community leaders, FH/M first identified all pregnant women and mothers of children under 24 months old in the project area. They then organized these mothers into blocks of 12 households and asked each group to elect a Care Group Volunteer (CGV) who would serve and promote behaviors with mothers throughout the project. Approximately 12 CGVs met together in a Care Group every 2 weeks with a paid project supervisor (called a “promoter”) to learn a child-survival health message or skill (Figure).

**FIGURE.**
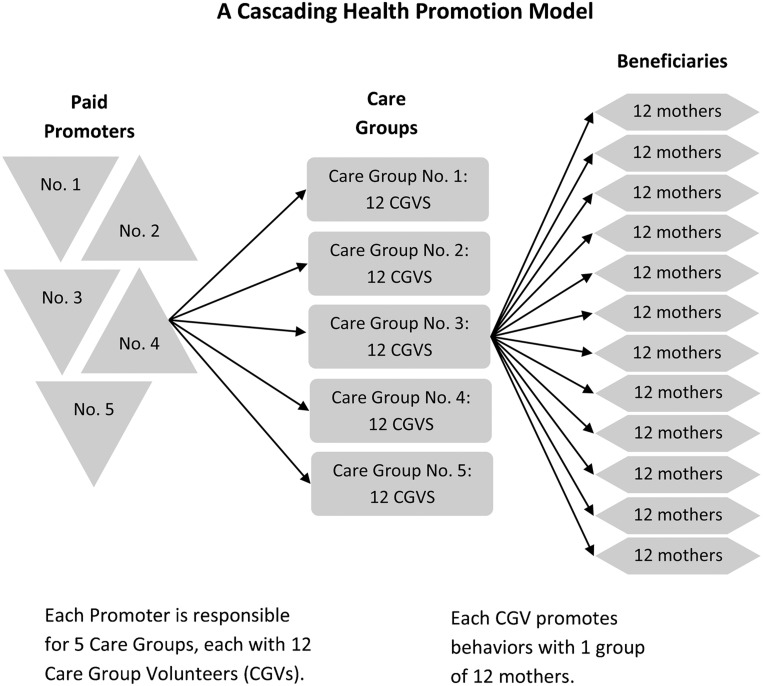
The Care Group Model as Implemented by Food for the Hungry in Sofala Province, Mozambique

Over the following 2 weeks, each CGV then met with the 12 pregnant women/mothers of children under 24 months old for whom she was responsible (from 12 nearby households) to share the messages and skills they had learned, using a flipchart with drawings (see supplemental material for a sample) to assist in conveying the behavior change messages (Box 1). CGVs met with these women either individually during one-on-one home visits or in small groups in their catchment area (with follow-up home visits to those who missed the small-group meetings). They delivered the entire set of messages over approximately a 2-year period. In the early intervention area, the messages were delivered twice.

**Box 1. Project Structure and Procedures**

**Staff Structure and Functions:**

- 1 Project Director, 1 Training Director, 1 Monitoring and Evaluation Coordinator, 5 Supervisors, and 65 Promoters
- Quarterly workshops to train promoters in key health-promotion messages
- Routine supervisory visits to the project communities to support Care Group meetings, for unannounced visits, and for mini-KPC survey data collection
- Technical support from Food for the Hungry/US staff virtually and through periodic field visits

**Care Group Model Structure and Functions:**

- Care Group Volunteers (approximately 1 for each 12 households) are selected by community leaders or beneficiary mothers in consultation with project field staff
- Promoter meets with a Care Group (composed of about 12 Care Group Volunteers) twice a month
- One new set of 2–3 health promotion messages are taught to the Care Group Volunteers at each meeting
- Each Care Group Volunteer promotes positive behaviors during the subsequent 2 weeks to the 12 mothers for which she is responsible, using the newly learned health promotion message

In general, all mothers received the same health promotion or nutrition message in a given 2-week period. However, when making home visits, CGVs also promoted messages related to the child's current problem (for example, promoting oral rehydration solution [ORS] when the child had diarrhea) or age-based needs (such as promoting exclusive breastfeeding in infants up to 6 months of age). Small-group meetings also provided an opportunity for encouraging peer support.

CGVs were trained in community-based integrated management of childhood illness (C-IMCI).^12^ Beyond that, the CGVs did not receive any additional training outside the community-level meetings held every other week, which usually lasted about 2 hours. During these meetings, CGVs also learned and sang songs based on the behaviors that they were promoting; shared their experiences (trouble-shooting with the promoter and other volunteers on how to persuade mothers to adopt the behaviors); and reported vital events. Gradually, as the CGVs learned from each other and the promoter, they became more confident and more effective in conveying these messages to their neighbors. CGVs received no cash, services, or in-kind incentives other than the 5 flipcharts used to transmit CG messages and one simple wraparound skirt decorated with health promotion messages (provided every 2 years).

International and local staff from FH/M developed the series of 24 messages collaboratively that CGVs delivered to promote good nutrition and to prevent and control diarrheal disease (Box 2). The choice of messages and behaviors promoted was influenced in particular by 2 types of rapid formative research studies.

**Box 2. Basic Content of Health Behavior Messages**

**Nutrition**

- Breastfeed infants immediately after birth and use colostrum.
- Exclusively breastfeed on demand until infants are 6 months old. Children should not be bottle-fed. Mothers should completely empty one breast before offering the infant the next one.
- Beginning at 6 months of age, children should be provided with appropriate complementary feeding, including iron-, iodine-, and vitamin-A-rich foods; breastfeeding should continue until the child is at least 24 months old.
- Complementary foods given to young children should be diverse, nutritionally dense, and thick enough to give a child the calories needed. Oil should be added to young children's food to ensure that it is energy-dense.
- Women should get voluntary counseling and testing and antenatal care. They should know their HIV status. Women with HIV infection should exclusively breastfeed only until the infant is 6 months old or until such time as exclusive replacement feeding becomes acceptable, feasible, affordable, sustainable, and safe. (We expected, and found, that replacement feeding was appropriate in very few cases in the project area.)
- Pregnant women and young children should consume iodized salt and marine products on a regular basis.
- Beginning at 6 months of age, children should receive vitamin A supplements every 6 months, and they should receive special treatment with vitamin A when they have measles and other severe infections.
- Mothers should receive vitamin A supplements within 6 weeks of delivery, or within 8 weeks if the mother is exclusively breastfeeding. (This was the WHO recommendation during the project period; WHO changed the recommendation in 2011.)
- Promotion of safe water and handwashing is an essential nutrition action. (See diarrhea section.)
- Cooked foods should not be stored without refrigeration for longer than 2 hours, and previously cooked food should be thoroughly reheated before eating.
- Children 0–23 months old should be taken to a health facility for growth monitoring on a monthly basis.
- Sick children should receive extra fluids, including more frequent breastfeeding, and they should be encouraged to eat soft, appetizing, favorite foods. Children should be given food more often during recovery.
- Children who are malnourished should be taken to a health facility for a medical examination.
- When a child will not eat or is losing weight, or when a mother has trouble breastfeeding, the Care Group Volunteer (CGV) or promoter trained in community-based integrated management of childhood illness (C-IMCI) should be contacted as soon as possible.
- Pregnant women should increase their nutritional intake, and they should take iron/folic acid supplements during pregnancy and lactation.

**Diarrhea**

- Give oral rehydration salts (ORS) or recommended home fluids to children with diarrhea. (Mothers were taught to make ORS.)
- Use the locally available water treatment product Certeza (or bleach, if Certeza is not available) to purify drinking water and safely store all drinking water used by the family to prevent cholera and other diarrheal diseases.
- Continue to feed and offer more fluids (including breastmilk) to children when they have diarrhea, and increase feeding immediately after illness.
- Children should be dewormed every 6 months.
- Wash hands with soap after defecation, before preparing meals, and before feeding children. Dispose of feces, including children's feces, safely. Use a “tippy-tap” (simple handwashing station made of commonly available materials, such as plastic bottles, that is not dependent on a piped water supply) or other water spreader to economize the amount of water needed for handwashing.
- Properly dispose of feces by constructing and using latrines to prevent diarrhea.
- When a child has diarrhea, the C-IMCI-trained CGV or promoter should be contacted immediately.
- When a child has signs of dehydration or general danger signs—for example, the child looks unwell; is not playing, eating, or drinking; is lethargic or has a change in consciousness; is vomiting everything; has a high fever or fast or difficult breathing—the child should be taken to a trained health worker immediately.
- Cooked foods should not be stored without refrigeration for longer than 2 hours.
- Avoid bottle feeding.
- Caregivers should follow the health care worker's advice about treatment, follow-up, and referral to government facilities for diarrhea.
- When a child has bloody or persistent diarrhea, seek care immediately from trained health care workers. (Dysentery is fairly rare in Sofala, but persistent diarrhea was on the rise at the time the project began.)

The first was a positive deviance analysis conducted in the project area in September 2004 (before program start-up) to determine the nutritional and child-care practices of mothers of well-nourished children compared with the practices of mothers of children with poor weight-for-age.^13^ Findings prompted the project to pay more attention to several key behaviors, such as letting the breastfeeding infant suck on the first breast until satisfied before offering the other breast and point-of-use (POU) water treatment in the home.

The second type of rapid formative research used to inform project planning was a series of Barrier Analysis studies of key health behaviors, including exclusive breastfeeding, handwashing with soap, and use of ORS.^14-15^ In these studies, mothers who were practicing a particular behavior (the “doers”) were compared with those who were not (the “non-doers”) to identify behavioral determinants (including barriers and enablers) of the behaviors studied. Results were then used to create or modify project activities and messages.

### Evaluation of the Project

We conducted baseline and endline Knowledge, Practices, and Coverage (KPC) surveys in both project areas, with randomly sampled households that had either pregnant women or mothers of young children (the beneficiaries of the project). The Area A baseline survey took place in February 2006, and the Area B baseline survey in February 2009. The endline surveys for both areas were conducted in June 2010.

The community collaborated at the outset of the project to identify all households with project beneficiaries (total of *T* households). From this list, we determined which respondents to interview by randomly starting with one household and then selecting every “nth” household (where *n* was determined by dividing *T* by 96).

In total, we interviewed approximately 100 mothers with children 0–11 months old and 100 mothers with children 12–23 months old for the baseline and endline surveys in each of Areas A and B of the project. We also weighed each “index” child whose mother was interviewed with a Salter spring scale, as well as 2–3 additional children in adjacent households. Thus, the total number of children weighed in the different survey rounds ranged from 569 to 620 (Table 1). The interviews were carried out by project staff members in sites for which they did not have direct program responsibility.

**Table 1.** Nutrition-Related Practices and Outcomes Among Care Group Project Beneficiaries, Selected Districts of Sofala Province, Mozambique, 2005–2010

Project Indicators	Area A – Early Implementation					Area B – Late Implementation (delayed by 2.5 years)				
	Baseline (2006)		Endline (2010)		Difference	Baseline (2009)		Endline (2010)		Difference
	n/N	%(95% CI)	n/N	%(95% CI)	% Difference, *P* Value*	n/N	%(95% CI)	n/N	%(95% CI)	% Difference, *P* Value*
**Nutritional Outcome**										
Children 0–23 m who are underweight (WAZ < −2.0 SD)	139/537	25.9	101/569	17.8	8.1,	168/620	27.1	89/569	15.6	11.5,
		(22.1–29.7)		(14.5–21.0)	< 0.001		(23.0–31.2)		(12.6–18.7)	0.001
**Feeding Practices (as reported by mother or caretaker)**										
Infants 0–5 m who were fed only breast milk in the last 24 hours	11/46	23.9	35/47	74.5	50.6,	25/45	55.6	37/46	80.4	24.9,
		(11.1–36.7)		(61.5–87.4)	< 0.001		(40.5–70.7)		(68.6–92.3)	0.010
Children 9–23 m who receive food other than liquids at least 3 times/day	34/109	31.2	87/115	75.7	44.5,	54/124	43.5	82/125	65.6	22.1,
		(22.4–40.0)		(67.7–83.6)	< 0.001		(34.7–52.4)		(57.2–74.0)	0.001
Children 6–23 m with oil added to their weaning food	47/130	36.2	126/145	86.9	50.7,	84/143	58.7	130/149	87.2	28.5,
		(27.7–44.5)		(81.3–92.5)	< 0.001		(50.6–66.9)		(81.8–92.7)	0.001
Children 6–23 m who have consumed at least one Vitamin A-rich food in the previous day	40/131	30.5	88/150	58.7	28.1,	80/157	46.5	102/150	68.0	21.5,
		(22.5–38.5)		(50.7–66.6)	< 0.001		(38.6–54.3)		(60.4–75.6)	0.001
Children 0–23 m with diarrhea in the last 2 weeks who were offered the same amount of, or more, food during the illness	22/68	32.4	35/42	83.3	51.0,	26/76	34.2	29/40	72.5	38.3,
		(20.9–43.8)		(71.6–95.1)	< 0.001		(23.3–45.1)		(58.0–87.0)	0.001
**Vitamin A Supplementation, Deworming, and Nutritional Monitoring**										
Children 12–23 m who have received one Vitamin A capsule in the past 6 months (card-confirmed or mother's report)	74/90	82.2	88/94	93.6	11.4,	80/101	79.2	94/98	95.9	16.7,
		(74.2–90.3)		(88.6–98.7)	0.015		(71.2–87.3)		(91.9–99.9)	0.001
Children 12–23 months who received deworming medication in the last 6 months (mother's report)	24/84	28.6	59/75	78.7	50.1,	36/96	37.5	67/73	91.8	54.3,
		(18.7–38.4)		(69.2–88.2)	0.001		(27.6–47.4)		(85.3–98.2)	0.001
Children 0–23 m who were weighed in the last 4 months (card-confirmed)	114/156	73.1	150/170	88.2	15.2,	115/172	66.9	131/158	82.9	16.1,
		(66.0–80.1)		(83.3–93.1)	0.001		(59.8–74.0)		(77.0–88.8)	0.001
**Diarrheal Disease (as reported by mother or caretaker)**										
Children 0–23 m with diarrhea in the last 2 weeks who received ORS and/or RHF	40/69	58.0	42/45	93.3	35.4,	49/79	62.0	38/43	88.4	26.3,
		(46.0–69.9)		(85.8–100.9)	0.001		(51.1–73.0)		(78.4–98.4)	0.002
Mothers of children 0–23 m who can correctly prepare ORS	77/177	43.5	166/196	84.7	41.2,	89/200	44.5	166/196	84.7	40.2,
		(36.1–50.9)		(79.6–89.8)	0.001		(37.6–51.4)		(79.6–89.8)	0.001
Mothers of children 0–23 m who report that they wash their hands with soap/ash before preparing food, before eating, after defecating, and after attending to a child who has defecated	2/199	1.0	100/198	50.5	49.5,	27/211	12.8	86/199	43.2	30.4,
		(0.1–334.0)		(43.3–57.7)	0.001		(8.6–18.1)		(36.2–50.4)	0.001
Mothers of children 0–23 m who report that they purify their water using any effective method (by boiling or using point-of-use water purification)	39/95	41.1	129/151	85.4	44.3,	26/211	12.3	135/153	88.2	75.9,
		(31.2–50.9)		(79.8–91.1)	0.001		(7.9–16.8)		(83.1–93.3)	0.001

Abbreviations: WAZ, z-score for weight-for-age; SD, standard deviation; ORS, oral rehydration solution; RHF, recommended home fluids.

* Statistical significance based on one-tailed Fisher exact test (based on difference in prevalence between endline and baseline results). *P* values < 0.05 were considered statistically significant. All *P* values were statistically significant.

The project also carried out periodic KPC “mini-surveys” (approximately every 6 months) in which we selected a random sample of households to assess coverage levels for specific indicators related to interventions that had been implemented in the previous 3–6 months. Project promoters conducted these interviews in adjacent supervisory areas other than their own.

Finally, in early 2010 we conducted focus group discussions with beneficiary mothers, CGVs, and promoters to better understand the degree to which the Care Group model was implemented as planned, the benefits perceived by the volunteers and beneficiaries, and other operational issues. Based on this, we developed and conducted 2 quantitative surveys in April 2010 to measure the outreach of CGVs—one with a random sample of 200 beneficiary mothers and 200 CGVs (100 from each project area) and the second with all 60 Care Group promoters in Areas A and B.

### Sample Sizes for Evaluation Surveys

Sample sizes for the household surveys were calculated to provide estimates of coverage in each of the 2 project areas based on a 95% confidence interval (CI) of +/− 10%, 90% power, and an estimated 20 percentage point difference between coverage levels at the time of the baseline and final evaluations. For a simple random sample, this required 103 respondents from each project area. For several indicators, however, the required sample size was considerably larger given that the expected amount of change (in percentage points) was lower. For example, to assess nutritional status, we needed a sample size of at least 586 children ages 0–23 months old to detect a statistically significant change from the projected 25% prevalence of undernutrition at baseline to the projected 18% at endline (with a 95% CI and 90% power).^16^

### Data Quality Assurance

Survey supervisors and investigators reviewed data forms for accuracy, consistency, and completeness. Data were entered in databases, which were reviewed for range and consistency. We used Epi Info™ (version 6.04d) for data entry, initial data analysis, and initial anthropometric analysis. A second round of independent data analysis was conducted by one of the authors (R.C.) using Stata (version 11.2) statistical software.

To clean the anthropometric data, we calculated the age of the index child. Then, we determined the difference between age stated by the mother and calculated age (per the date of birth). If the difference was more or less than 2 months, we removed the respondent from the anthropometry dataset. We also removed data that were extreme outliers, for example, data for children with a negative calculated age or with biologically unreasonable anthropometric data (as flagged by Epi Info).

Data were collected as part of ongoing project monitoring and evaluation activities. The Johns Hopkins Bloomberg School of Public Health Institutional Review Board reviewed the participation of its staff in the project and determined that their activities did not require human subjects review.

### Data Analysis

We calculated proportions with 95% confidence intervals for the coverage of child-survival interventions and for underweight children, accounting for clustering of anthropometric measures of undernutrition. Mothers were selected independently for interviewing, so there was no need to account for clustering of their responses. We compared baseline and endline indicators to determine the magnitude of the difference and whether the difference, if observed, was statistically significant. The prevalence of undernutrition in the project areas was determined by comparing anthropometric measurements with WHO child growth standards established in 2006.^17^

### Role of the Funding Source

The project was funded by the Child Survival and Health Grants Program of the United States Agency for International Development (USAID) and by Food for the Hungry. USAID reviewed the project design but played no role in the data collection, analysis, or interpretation, or in the decision to submit this paper for publication.

## RESULTS

### Findings From CGV Surveys

The surveys to measure outreach of CGVs revealed that 44% of CGVs were elected by their mother groups, while 55% were selected by either the community leader or promoter. Mothers may have been more likely to select a “hub” in their social network, which could have facilitated behavior change, since some nutrition and health behaviors are transmitted through social networks,^18-19^ and some behaviors “cluster” in social networks.^20^

The surveys also found that CGVs who were elected by their peers were 2.7 times more likely to serve for the entire length of the project (Odds Ratio [OR] = 2.7, 95% CI = 1.19–5.99; *P*<0.01). The average walking time between CGVs' houses and the most distant house of the women that they served was 15 minutes, and between the CGVs' houses and the community location used for their biweekly training was 17 minutes.

The surveys found that 30% of CGVs worked mainly or exclusively through home visits, while 70% worked mainly with small groups, with follow-up home visits to mothers who missed the small-group meetings. The surveys also found that 68% of CGVs reported that home visits lasted less than one hour, and 82% reported that small-group meetings lasted at least one hour. Newly pregnant women and mothers with young children who moved into the project area were put under the charge of existing CGVs. In order to ensure a continued reasonable workload for volunteers, once a child turned 24 months of age, the CGV was no longer expected to visit the mother in her home. However, the mother was allowed to continue attending neighborhood small-group meetings with the other pregnant women and mothers of young children.

CGVs achieved a high contact level with mothers in their catchment areas over the life of the project. For example, 3 short KPC household surveys, conducted at 3 separate times during 2007, documented that an average of 91% of mothers with children ages 6–23 months, and 94% of mothers with children ages 0–5 months, had received a visit by the CGV in the previous 2 weeks. In addition, turnover of volunteers was low (5% annually), and less than 1% of CGVs quit due to a stated lack of material incentives.

The CGV surveys found that respect gained from other people in their social network—and seeing the results of the project—were key motivators for volunteers. All surveyed CGVs reported that they were more respected by other women in their community because of their participation as a CGV, and 64% reported being more respected by community leaders. In addition, 61% and 48% reported being more respected by their husbands and parents, respectively, and 25% by health facility staff.

It is interesting to note, as well, that the proportion of CGVs who had accepting attitudes toward domestic abuse was only 3% at the end of the project compared with 24% of the beneficiary mothers whom they served. The CGV surveys also found that 65% of CGVs had communicated with health facility staff about their child-survival activities and topics at least once in the previous year, and 68% had communicated with their community leaders at least once in the previous 3 months.

### Findings From KPC Household Surveys

Baseline and endline KPC household surveys indicate several statistically significant positive improvements (*P* = 0.01 or less) in nutrition-related behaviors (with increases as high as 52 percentage points), including with exclusive breastfeeding, provision of solid foods at least 3 times per day for children 9–23 months, adding oil to weaning foods for children 6–23 months, consumption of at least one vitamin-A rich food during the previous day in children 6–23 months, and provision of the same amount of food or more among children with diarrhea during the previous 2 weeks (Table 1).

Receipt of vitamin A supplementation and deworming medication during the previous 6 months, weighing of children during the previous 4 months (card-confirmed), improvements in mother's knowledge of how to prepare ORS for children with diarrhea, and use of ORS for children with diarrhea during the previous 2 weeks all demonstrated statistically significant differences (*P*<0.01) between baseline and endline indicators. Baseline levels of receipt of vitamin A supplementation were much higher than for other baseline indicators, but these levels also improved over the course of the intervention in both early intervention (Area A, *P* = 0.015) and delayed intervention (Area B, *P*<0.001) areas.

The following household behaviors promoted by the project all showed statistically significant increases in both Areas A and B when comparing endline with baseline measures (Table 1):

- Improved nutrition (exclusive breastfeeding, appropriate complementary feeding, adding oil to weaning foods, and ingestion of vitamin A rich foods)
- Diarrhea treatment (provision of increased fluids for childhood diarrhea, continued feeding with diarrhea, and knowledge of how to prepare ORS correctly)
- Diarrhea prevention (handwashing and point-of-use water purification in the home)

Based on mini-KPC surveys carried out in both intervention areas, a rapid uptake of these behaviors was achieved (data not shown). In Area B, all indicators showed statistically significant improvements by the time of the endline evaluation less than 2 years after project initiation in that area.

Mothers in the project areas improved their treatment and prevention of diarrhea over time.

#### Changes in Childhood Nutritional Status

In the early intervention area (Area A), the percentage of children 0–23 months old with global undernutrition declined significantly by 8.1 percentage points (*P*<0.001) over a 5-year period (Table 1), from 25.9% at baseline (95% CI = 22.2%–29.6%) to 17.8% at endline (95% CI = 14.6%–20.9%). Global undernutrition was defined as weight-for-age with a z-score of less than 2 standard deviations below the international standard median/mean.

Undernutrition among children 0–23 months old declined significantly and rapidly in the project areas, at about 4 times the rate of decline among children 0–59 months old nationwide.

In the delayed intervention area (Area B), over only a 16-month period, the prevalence of global undernutrition declined significantly by 11.5 percentage points (*P*<0.001), from 27.1% (95% CI = 23.6%–30.6%) to 15.6% (95% CI = 12.6%–18.6%). The average annual decline in Area B was estimated by assuming that the baseline data for this area reflected 2005 values and that the project was conducted over a full 5-year period. Even under these conservative assumptions, the annual rate of decline among children 0–23 months old in Areas A and B was approximately 4 times the rate of decline among children 0–59 months old nationwide in Mozambique (Table 2).

**Table 2.** Average Annual Rate of Decline in Undernutrition in the Care Group Mozambique Project Areas Compared With Mozambique Nationwide

Location	Age group of children^a^	% of children < 2 SD below the standard median/mean of weight-for-age		% Difference	No. of years (endline – baseline)	Avg. annual rate of decline
		Baseline (dates)	Endline (dates)			
Project Areas	0–23 m	26.5%(Feb 2006)	16.7%(Jun 2010)	9.8%	4.4	2.2%
Nationwide^b^ (DHS and UNICEF/MICS)	0–59 m	20.0%(2003)	18.0%(2008)	2.0%	5	0.4%
Nationwide^b^ (DHS)	0–59 m	20.0%(2003)	14.9%(2011)	5.1%	8	0.6%

Abbreviations: SD, standard deviation; DHS, Demographic and Health Surveys; MICS, Multiple Indicator Cluster Survey.

a Comparable national data for children 0–23 months old from the DHS and MICS surveys are not available.

b Nationwide data are from the 2003 and 2011 DHS^21-22^ and the 2008 UNICEF/MICS.^23^ The 2003 DHS reported an undernutrition level of 24.6% using earlier WHO nutritional standards. The 2008 UNICEF/MICS survey recalculated the 2003 DHS numbers, shown here, using the WHO 2006 nutritional standards.

### Project Costs and Other Project Benefits

Total project costs were US$3.0 million, of which $2.5 million was provided by the USAID Child Survival and Health Grants Program and $0.5 million by Food for the Hungry (Table 3). This represents an average cost of $0.55 per capita per year (when considering the entire project population of 1.1 million people) and $2.78 per beneficiary per year (when considering the 219,617 mothers with children ages 0–23 months in the project areas as the project beneficiaries).

**Table 3.** Care Group Project Costs and Number of Beneficiaries, Selected Districts of Sofala Province, Mozambique, 2005–2010

Project Site, Dates	Total Project Costs	Total Population	Total Cost per Capita per Year	No. of Beneficiaries	Total Cost per Beneficiary per Year
Area A,^a^ Oct 2005–Sep 2010 (5 years)	$2,026,191	462,000	$0.88	92,239	$4.39
Area B,^b^ Mar 2009–Sep 2010 (1.6 years)	$997,975	638,000	$0.97	127,238	$4.90
Total Project	$3,024,166^c^	1,100,000	$0.55	219,617	$2.78

All dollar amounts expressed in US$.

a Area A included Caia, Chemba, Manga, and Marringue Districts.

b Area B included Dondo, Gorongosa, and Nhamatanda Districts.

c Includes contributions of $2,499,901 from USAID to Food for the Hungry and $524,166 from Food for the Hungry unrestricted funds.

The Care Group project cost, on average, only US$0.55 per capita per year.

Findings from the CGV surveys found that the 4,095 CGVs donated a total of 2.4 million volunteer hours serving their neighbors. The project carried out other important maternal and child health interventions, including promotion of birth spacing, antenatal care, and facility-based deliveries and attendance at birth by appropriately trained personnel; provision of appropriate newborn care; use of insecticide-treated bed nets; recognition of serious childhood illness and referral to facility-based care for children with danger signs; and promotion of HIV/AIDS awareness. The project's impact on these indicators, most of which showed similar statistically significant gains, will be reported in subsequent publications.

## DISCUSSION

This project evaluation demonstrates that an innovative and low-cost behavior change strategy using Care Groups (a volunteer, peer-educator model) achieved major improvements in nutrition-related household behaviors among a population of 1.1 million people in rural Mozambique, an area where diarrhea and malnutrition are major contributors to child mortality. The behaviors on which the project focused are known to be influential in improving child health and reducing under-5 mortality.

The evaluation also demonstrates that the Care Group model achieved a statistically significant decline in the percentage of children ages 0–23 months with global undernutrition. The rate of reduction in both project areas combined was 4 times that in Mozambique nationwide, according to national surveys done during approximately the same time period. The project achieved these results by relying on volunteers to visit households every 2 weeks over a 5-year period at a cost of only $0.55 per capita; no food supplementation was provided.

To our knowledge, this is the first report in the peer-reviewed literature of a project documenting input, process, outcome, and impact measures and providing evidence of improving child nutrition at scale (in a population of more than 1 million people) as a result of a behavior change strategy that did not involve food distribution.*

A recent review from the Bill & Melinda Gates Foundation pointed to the lack of practical knowledge on how best to change nutrition-related behaviors, such as breastfeeding behaviors, as well as to the lack of tools to measure the impact of existing interventions on a person's nutritional status.^24^ The review concluded that scale up of proven and affordable nutrition interventions targeted at pregnant women and children up to 2 years of age is urgently needed to reduce nutrition-associated death and disability.

This paper responds to a critical need and points to a promising approach for implementing and evaluating nutrition programs that merits widespread field application and rigorous evaluation.

While the delayed intervention area (Area B) received the intervention for a considerably shorter period of time (16 months versus 53 months), it showed a greater decrease in undernutrition prevalence than the early intervention area (Area A). There are several possible explanations for this finding. First, project leaders and supervisors had become more effective and efficient in starting up and implementing the interventions based on their experiences with the early intervention area. Second, intervention effectiveness may peak initially and then decline to some degree over time. In Area A, for instance, there were rapid increases in intervention coverage during the first year of project functioning. The project maintained high levels of coverage over the life of the project, but there was some decline over time. Thus, the impact evaluation in Area B may have been conducted just as coverage of interventions was at its peak. In Area A, there may have been a decline in coverage by the time of the final evaluation. These issues will be explored in further analysis of the data in a forthcoming publication.

The fact that the levels of certain baseline indicators in Areas A and B were significantly different merits exploration. Baseline indicators in Area B were measured 36 months after they were measured in Area A. Differences between Areas A and B were statistically significant for 3 indicators: level of exclusive breastfeeding, addition of oil to weaning foods, and consumption of vitamin-A rich foods. For all 3 indicators, levels were higher at baseline in Area B than in Area A (Table 1). These findings raise the possibility that a secular trend independent of the project may have been present that could account for the changes observed in nutritional status.

We think this is unlikely because baseline levels of undernutrition did not differ in Areas A and B. In addition, Food for the Hungry had operated nutrition programs in several Area B districts between 1997 and 2001, which could have contributed to the more favorable indicator levels.

Several design characteristics of the model may be responsible for the model's success. It may be instructive for policy makers, program planners, and practitioners to consider these characteristics when designing and operationalizing nutrition projects in diverse systems settings and at different scale:

1. **Reaching targeted households on an ongoing basis through peer-to-peer education:** Empowering women to convey critical and relevant health and nutrition messages to their neighbors and ensuring that they reach all targeted mothers every 2 weeks provides a powerful platform for behavior change.
2. **Engaging beneficiaries in choosing peer educators:** Beneficiary mothers elected many of the CGVs who served them (44%), making the CGVs—the principle agents of behavior change—more likely to be “hubs” in the beneficiaries' social networks. Recent studies on behavior change show that some behaviors cluster and spread through social networks and that people who are better connected with others are more likely to influence the behavior of others in their networks.^18-20^
3. **Organizing beneficiaries into small, interactive groups that meet often and have close linkages with community leaders and health facility staff:** Stronger social capital results in the development of new norms about behavior compliance and participation in using health services among network members, provides information and knowledge to individuals in the group, and creates trustworthiness.^25^ The Care Group methodology connected pregnant women and mothers, who may have had little or no linkages with community resources, to CGVs on a regular basis. The CGVs, in turn, were linked with community leaders and health facility staff and served as a bridge between the pregnant women and mothers they served and the resources and knowledge held by the community leaders and health facility staff. Despite the fact that mothers, health facility staff, and community leaders may have infrequent contact with one another, important information can flow through the CGVs, who were already key links and hubs in the social network before becoming a CGV and whose work as a CGV further strengthens these linkages.^26^
4. **Keeping workloads of volunteers minimal to avoid overburdening them and ensure they cover beneficiaries well with their assigned tasks:** By using 4,095 volunteer workers, the project was able to cover 90% of almost 50,000 beneficiary households every other week. Keeping workloads and travel times minimal for CGVs almost certainly contributed to a 95% annual retention rate of volunteers. The high level of repeated contact between these peer educators and the small number of mothers each one served may have made it easier for them to work through barriers to behavior change. It also probably helped them develop the relationships necessary for behavior change to happen. Peer-education models that do not reach high levels of coverage in the community sometimes fail to show results.^27^ Changes in *community-wide* nutritional practices are often not seen when population coverage of a program is low even if the program affects change at the *individual* level.
5. **Conducting rapid formative research to select key behaviors and their determinants**: Some of the largest changes in behavioral indicators were in the behaviors that were uncovered or explored during rapid formative research. For example, prevalence of exclusive breastfeeding during the first 6 months of life and of handwashing with soap/ash each increased by 50 percentage points (Table 2). Implementers should allocate appropriate levels of resources for these rapid studies† and use results to modify activities and messages.
6. **Empowering women by giving them active, volunteer roles in the project**: By using volunteers, the project was able to leverage more than 2.4 million hours of volunteer service. Rather than the volunteer duties being a burden on these women, 96% of the volunteers continued to volunteer each year; most identified several benefits of serving as volunteers, such as increased respect by husbands, peers, family members, community leaders, and health facility staff; and many expressed great enthusiasm during the final evaluation for continuing their health promotion work after the project ended. Other studies have found that the benefits to volunteers may remain long after they relinquish their volunteer role, and people who volunteer frequently are more likely to report higher life satisfaction than non-volunteers.^28-29^

Keeping workloads of Care Group Volunteers light contributed to a very low turnover rate.

Qualitative data from focus group discussions at the end of the project provide further evidence that behaviors did in fact change, that mothers noted improvements in their children's nutritional status, and that the frequency of child death had declined.

Two previous reports have noted the effectiveness of the Care Group approach in improving child health. An evaluation of a child survival project in southern Mozambique (Gaza Province), using a similar Care Group methodology to promote child-survival interventions related to malaria, diarrhea, nutrition, and immunizations, demonstrated marked and statistically significant increases in coverage of the project's interventions.^30^ A separate, independent mortality impact assessment carried out for this same project found a 42% decline in under-5 mortality.^27^ The baseline project survey did not include a measure of nutritional status, so change in nutritional status was not assessed. A similar Care Group child survival project in Cambodia achieved high coverage of child-survival interventions rapidly, with a decline in the under-5 mortality rate from 129 per 1,000 live births to 35 in 5 years.^31^

Scaling up the Care Group model should be considered in rural areas with elevated under-5 mortality and high levels of child undernutrition. Governments could scale up the approach quickly and at a similar cost per capita either by contracting with NGOs to implement the model or by hiring supervisory staff to provide the necessary leadership and technical support and assigning MOH staff to work directly with Care Groups in the manner that Food for the Hungry did. We favor the former approach because contracts with NGOs could be readily linked with performance—a process that is more difficult to implement when MOH staff provide the services directly. Concern Worldwide (an international NGO) is currently conducting a randomized trial in Burundi to compare the more traditional Care Group model where NGO staff members serve as promoters with an “integrated” model where MOH staff members serve as promoters.^32^

Scaling up the Care Group model would require some intensive training and orientation of key staff at the outset. It would also require a schedule of phasing in the program in various areas of a country and adjustments to the scaling-up process based on ongoing program evaluations. In the Mozambique context, training protocols, educational messages, and teaching materials (such as flipcharts to use during home visits) have already been developed. Other countries would need to adapt these resources to their particular settings since nutritional messages are context specific. We also recommend a rigorous independent evaluation with any scaling-up activities of the Care Group model because such rigorous evidence is lacking.

### Limitations of the Study

Evaluation of the project had certain limitations. First, we did not have an independent and separate monitoring and evaluation component; survey data were collected by project field staff. However, field staff collected data in project areas where they did not normally provide field supervision, so we believe this should mitigate any potential bias in the data collection process.

Selection of survey samples was based on project implementation data, and they were not selected through an independent method. This could have excluded certain geographic areas or neighborhoods from the sampling frame and potentially biased the results. However, we think this is highly unlikely since we worked closely with the communities at the outset of the project to ensure that all beneficiary households in the community were included in the sampling frame and program implementation.

Conducting the baseline and final anthropometric surveys during different months of the year could potentially lead to declines in nutritional status based mostly on seasonal patterns in nutritional status. However, if anything, actual reductions in malnutrition may have been greater than observed since the baseline was conducted during the first harvest period in Sofala Province (February 2006),^33^ and the final was conducted a month after the end of the first harvest (June 2010).^34^

Another limitation is the lack of a comparison area. However, the presence of national DHS survey data provide further evidence that the observed changes are quite likely to have been produced by the project intervention. Still, comparing rates of decline in childhood undernutrition in the project areas with national data from the DHS is less than ideal for several reasons. First, national data are for children 0–59 months old while data from the project areas are for children 0–23 months old. Second, we do not know the degree to which comparison of these 2 areas is appropriate. Nonetheless, these are the best data available at present for comparison. If the WHO nutritional standards had not changed in 2006 (from being based on data from children in the United States to children around the world raised in optimal circumstances), it would have been possible to assess changes in undernutrition among children 0–59 months of age in Sofala Province, as well as for children 0–23 months of age nationally. This would have provided additional evidence to judge whether the rate of decline in undernutrition in the project areas occurred at a more rapid rate than in other areas.

Finally, measuring height along with weight would have provided important information about the nutritional status of children in the study.

This project was not set up as a research study, and funds were not available to overcome the limitations described above. But even with these limitations, we consider that the entire set of findings provides persuasive and plausible—albeit not definitive—evidence that the Care Group Project as implemented in Sofala Province by Food for the Hungry was effective in improving childhood undernutrition at scale. Given the scarcity of such evidence in the nutrition field, we think the findings presented in this paper justify more rigorous trials of the effectiveness of the Care Group approach.

## SUMMARY AND CONCLUSION

The Care Group child-survival project described in this paper achieved high levels of regular and frequent peer-to-peer contact with pregnant women and mothers of children 0–23 months old in a challenging environment with high levels of under-5 mortality and malnutrition. Coverage of key interventions for preventing and treating childhood diarrhea and promoting good nutrition expanded dramatically in the project areas. The resulting rate of decline in childhood undernutrition was 4 times that for Mozambique nationwide. The project achieved these results at a cost readily affordable to very poor countries—only US$0.55 per capita. These findings, together with other published results on the effectiveness of the Care Group model,^30-31^ provide a growing evidence base that supports the importance of this model in accelerating progress toward reducing under-5 mortality in Africa and other priority countries where progress has been lagging. The model points to the kinds of strategic shifts that these countries will need to make—both in terms of reorienting service delivery and building partnerships with NGOs for community-based service delivery. Further prospective assessments of the Care Group approach using more rigorous methodologies are needed.

## Footnotes

* We searched PubMed using the following search terms: malnutrition OR stunting OR wasting with community-based nutrition programs OR community-based program OR community involvement OR community participation OR community programs, and similar terms.

† Practitioners can view and share their Barrier Analysis and Doer/Non-Doer studies at: www.fsnnetwork.org/behavior-bank
